# New insights into the structure and function of microbial communities in Maxwell Bay, Antarctica

**DOI:** 10.3389/fmicb.2024.1463144

**Published:** 2024-09-04

**Authors:** Zheng Wang, Zhiwei Gao, Yong Yu, Huirong Li, Wei Luo, Zhongqiang Ji, Haitao Ding

**Affiliations:** ^1^Antarctic Great Wall Ecology National Observation and Research Station, Polar Research Institute of China, Ministry of Natural Resources, Shanghai, China; ^2^Key Laboratory for Polar Science, Polar Research Institute of China, Ministry of Natural Resources, Shanghai, China; ^3^School of Oceanography, Shanghai Jiao Tong University, Shanghai, China; ^4^Key Laboratory of Marine Ecosystem Dynamics, Second Institute of Oceanography, Ministry of Natural Resources, Hangzhou, China

**Keywords:** diversity, distribution, ecosystems, environmental factors, potential function

## Abstract

The microbial communities inhabiting polar ecosystems, particularly in Maxwell Bay, Antarctica, play a pivotal role in nutrient cycling and ecosystem dynamics. However, the diversity of these microbial communities remains underexplored. In this study, we aim to address this gap by investigating the distribution, environmental drivers, and metabolic potential of microorganisms in Maxwell Bay. We analyzed the prokaryotic and eukaryotic microbiota at 11 stations, revealing distinctive community structures and diverse phylum dominance by using high-throughput sequencing. Spatial analysis revealed a significant impact of longitude on microbial communities, with microeukaryotes exhibiting greater sensitivity to spatial factors than microprokaryotes. We constructed co-occurrence networks to explore the stability of microbial communities, indicating the complexity and stability of microprokaryotic communities compared with those of microeukaryotes. Our findings suggest that the microeukaryotic communities in Maxwell Bay are more susceptible to disturbances. Additionally, this study revealed the spatial correlations between microbial communities, diversity, and environmental variables. Redundancy analysis highlighted the significance of pH and dissolved oxygen in shaping microprokaryotic and microeukaryotic communities, indicating the anthropogenic influence near the scientific research stations. Functional predictions using Tax4Fun2 and FUNGuild revealed the metabolic potential and trophic modes of the microprokaryotic and microeukaryotic communities, respectively. Finally, this study provides novel insights into the microbial ecology of Maxwell Bay, expanding the understanding of polar microbiomes and their responses to environmental factors.

## 1 Introduction

Microbes are essential in natural ecology (Konopka, 2009; Tiedje et al., 2022) and drive the Earth’s biogeochemical cycles (Cavicchioli et al., 2019; Falkowski et al., 2008). Polar regions, with their significant influence on the Earth’s ecology, are a focal point of research attention (Ji et al., 2022). Ecological changes in these areas markedly affect global ecosystems, particularly those in Antarctica (Barrett et al., 2006; Cowan et al., 2014). The Antarctic Ocean microbiome is crucial in the biochemical cycles of the region (Coutinho et al., 2021; Niederberger et al., 2019), as marine microorganisms participate in almost all biochemical reactions in the ocean and significantly impact the biological carbon (Moran et al., 2022), nitrogen (Hutchins and Fu, 2017), and sulfur (Li et al., 2023) cycles in marine ecosystems.

Generally, community variation along geographical gradients is a well-known ecological trend (Leroux, 2018; Lurgi et al., 2020), and fine-scale analyses may provide insights into the community dynamics and functions of the ocean microbiome (Leigh et al., 2018; Xu et al., 2022). Previous studies have shown that the microbial diversity is affected by environmental and biological factors on large and local scales (Buschi et al., 2023; Toledo et al., 2023), and total beta diversity and species turnover of bacteria can be determined using spatial, environmental, and biotic variables (Wu et al., 2020). Therefore, understanding the distribution and diversity of the microbiome in Antarctica is crucial for elucidating the intricacies of these ecosystems. Microbial communities respond to environmental gradients, and studying their spatial patterns provides valuable insights into the factors influencing their composition and function.

Maxwell Bay, nestled in the Antarctic, is a microcosm of Antarctic microbial communities (Rego et al., 2020). Its geographical location within the Southern Ocean, surrounded by King George Island, a large, ice-free and biodiverse area in maritime Antarctica (Bello et al., 2022), provides a unique setting for studying the adaptability and resilience of the microbiome to extreme environments (Zhang et al., 2018). In addition, Maxwell Bay has a high density of scientific stations where most biological studies are conducted (Amaro et al., 2015). Studies have been conducted on bacterial and eukaryotic communities in specific locations within Maxwell Bay, including the Great Wall, Ardley (Liu and Jiang, 2020; Luo et al., 2015; Zeng et al., 2014), and Marian Coves (Kim et al., 2020), however, comprehensive investigations of the overall microbial distribution in the bay are lacking. The microbial driving factors are unclear, and their potential ecological functions remain unexplored.

Therefore, in this study, we collected samples from 11 stations in Maxwell Bay. And the objectives of this study were to (1) characterize the distribution patterns of microprokaryotic and microeukaryotic communities; (2) investigate the impact of environmental parameters on microbial communities; (3) potential functions of the microbiome in Maxwell Bay. To some extent, our findings might enhance the understanding of the microbial distribution patterns and their impact on the Antarctic and provide valuable insights for global ecosystems and environmental management and the assessment of global climate change.

## 2 Materials and methods

### 2.1 Sample collection

**Figure 1:**
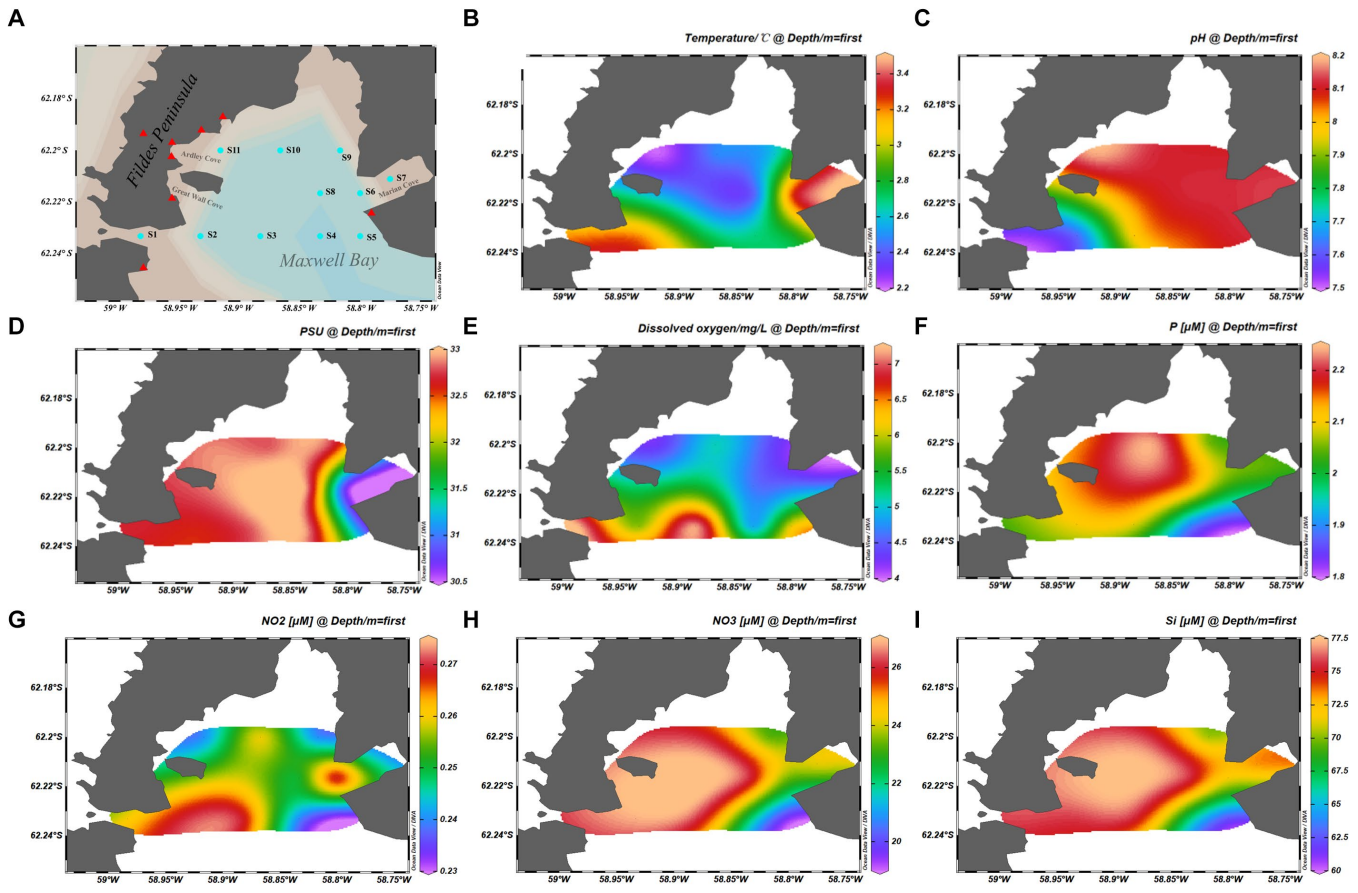
Map showing the samples information. **(A)** Geographical location of the stations from which samples were collected. **(B)** Temperature of samples. **(C)** pH of samples. **(D)** Practical salinity units of samples. **(E)** Dissolved oxygen of samples. **(F)** Phosphate concentration of the samples. **(G)** NO_2_^−^ concentration of the samples. **(H)** NO_3_^−^ concentration of the samples. **(I)** Silicate concentration of the samples. The cyan dots represent the sample stations, and the red triangles represent the scientific research stations.

Eleven samples were collected from Maxwell Bay in January 2017 during the Chinese 33th Antarctic research expedition. The sample locations are shown in Figure 1A. Samples were collected from surface water at all stations.

### 2.2 Physical and chemical composition of seawater

The physical and chemical properties of the analyzed samples are summarized in Figures 1B–I. Water temperature and salinity were measured using an YSI Model 30 instrument (Yellow Springs Instruments, Yellow Springs, OH, United States). Nutrients, including nitrite (NO_2_^−^ and NO_3_^−^), silicate (SiO_3_^2−^), and phosphate (PO_4_^3−^), were measured spectrophotometrically with a continuous flow auto-analyzer Scan++ (Skalar, The Netherlands) after filtering seawater through 0.45-μm cellulose acetate membrane filters (Whatman) (Luo et al., 2015). Dissolved oxygen (DO) concentration was determined using a previously described method (Strickland and Parsons, 1972).

### 2.3 DNA extraction, qualification, and sequencing analysis

Surface seawater (1 L) from each station was collected and pre-filtered through a 20-μm mesh sieve to remove most mesozooplankton and large particles and subsequently directly filtered through a 0.2-μm pore size nucleopore membrane filter (Whatman) (Zhang et al., 2022). The filters were frozen at −80°C in cetyltrimethylammonium bromide buffer until laboratory experiments were performed. DNA was extracted as previously described (Luo et al., 2009). The V3-V4 region of the 16S rRNA bacterial gene was amplified using the universal primers 338F (5′-ACTCCTACGGGAGGCAGCAG-3′) and 806R (5′-GACTACHVGGGTWTCTAAT-3′) (Wang et al., 2021). For the eukaryotic microbiota, the 18S rRNA gene was amplified using the primers SSU0817F (5′-TTAGCATGGAATAATRRAATAGGA-3′) and 1196R (5′-TCTGGACCTGGTGAGTTTCC-3′) (Yi et al., 2019). The primers contain barcode sequences unique to each sample. The PCR products were purified (Ren et al., 2015) using a PCR purification kit (Takara, Dalian, China), and their contents were measured using a Thermo Scientific NanoDrop8000 UV–vis spectrophotometer (NanoDrop Technologies, Wilmington, DE, United States). The barcoded PCR products were merged into equimolar quantities and subjected to high-throughput sequencing using a MiSeq benchtop sequencer for 2 × 300 bp DNA sequencing (Illumina, San Diego, CA, United States) at Majorbio, Ltd. (China). Microprokaryotes and microeukaryotes operational taxonomic unit (OTU) representative sequences were classified taxonomically by blasting against the Silva 138 database (Quast et al., 2013).

### 2.4 Sequence processing

All raw MiSeq-generated sequences were processed using QIIME (version 1.8) (Caporaso et al., 2010). High-quality sequences were obtained by removing sequences with ambiguous bases of >2, homopolymers of >10, primer mismatches, and average quality scores of <50 bp. Chimeras were removed using UCHIME software (Edgar et al., 2011). Subsequently, the trimmed sequences were clustered into OTUs with 97% similarity (Edgar, 2010), and the Shannon index, Simpson’s index (1-D), and Chao1 estimator values were calculated using UCLUST (version 1.2.22) at the OTU level (Edgar, 2013; Simpson, 1949; Tao et al., 2014).

### 2.5 Statistical analyses

Pearson’s correlation coefficient, linear fitting, and chord diagrams were conducted using OriginPro 10.05. The R program (version 4.2.3) was used to perform heat mapping, variation partitioning analysis, redundancy analysis (RDA), Principal Coordinates Analysis (PCoA), and the Monte Carlo permutation test. In the heat map, metabolic pathways and trophic modes were transformed using *z*-scores. Community similarity was determined using Bray–Curtis distance matrices (Beals, 1984) with 999 permutations. Analysis of similarities (ANOSIM) with 999 permutations was performed by using the vegan package in R to determine significant differences between samples based on Bray–Curtis distance matrices (Bokulich et al., 2014). Tax4Fun2 and FUNGuild software were used to predict microprokaryotic (Wemheuer et al., 2020) and microeukaryotic (Polme et al., 2020) functions, respectively. Visualizations of microbial network analysis were performed using the Gephi software (version 0.9.1) (Wang et al., 2019).

## 3 Results

### 3.1 Microbial community structure and diversity

**Figure 2:**
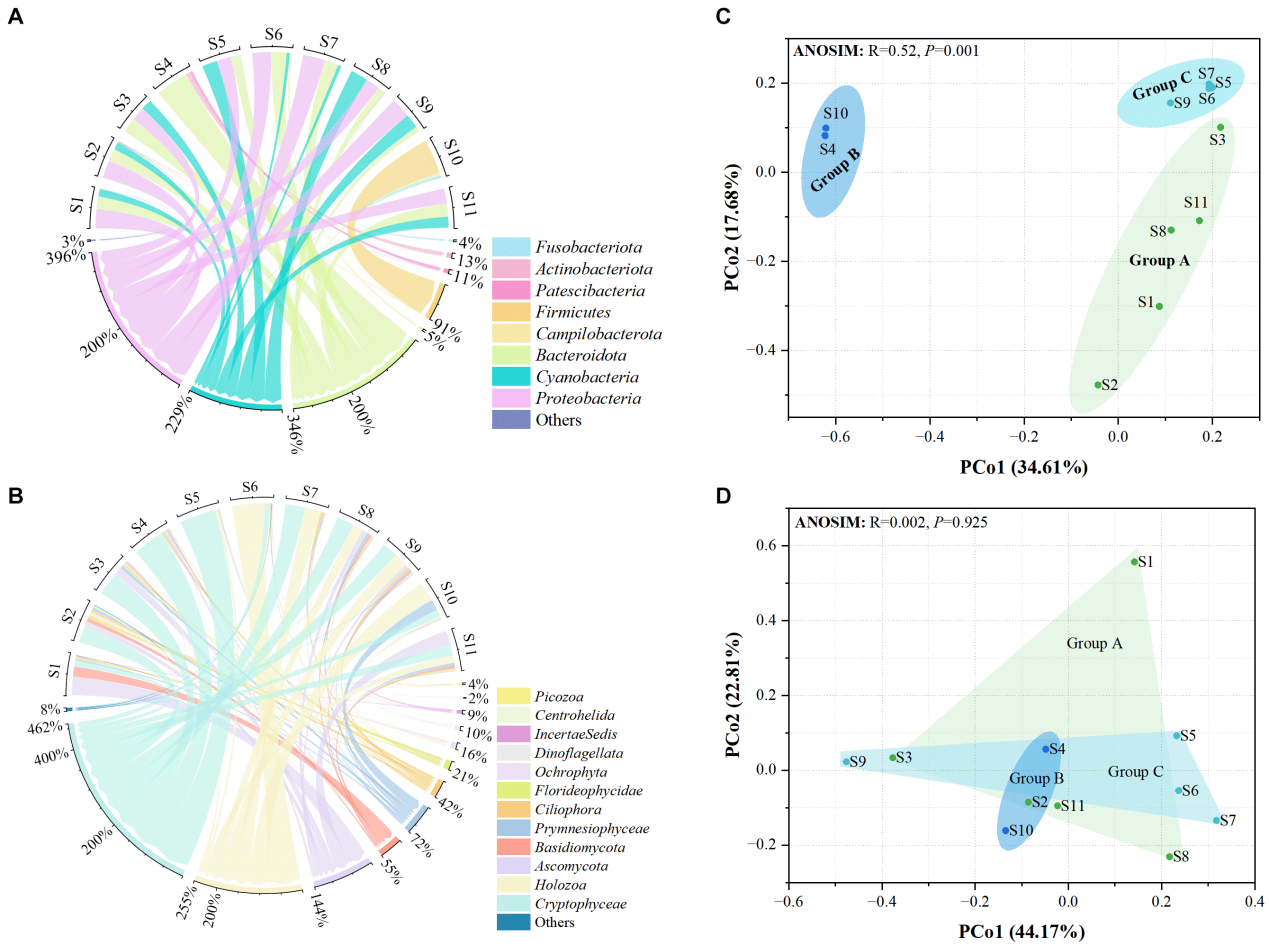
Distribution of the microbiota at the 11 stations. **(A)** Prokaryotic microbiota. **(B)** Eukaryotic microbiota. Only phyla with an average abundance of >1% are indicated. Phyla with less than 1% abundance are combined and shown in the “others” category. **(C)** Principal Coordinates Analysis (PCoA) of the prokaryotic microbiota. **(D)** PCoA of the eukaryotic microbiota. Significance was determined using ANOSIM.

A total of 425,993 and 410,151 high-quality sequences were obtained for prokaryotic and eukaryotic microbiota, respectively. After quality control of all samples, 481 and 355 OTUs were obtained for microprokaryotes and microeukaryotes, respectively, with 97% similarity. In all, 12 prokaryotic and 32 eukaryotic microbial phyla were identified in all the samples. Only 8 and 12 prokaryotic and eukaryotic microbial phyla were dominant (average relative abundance of >1%). Their relative abundances are shown in Figures 2A,B. In the prokaryotic microbiota, the predominant phyla were *Bacteroidota*, *Cyanobacteria*, and *Proteobacteria* (relative abundances ≥10%) in S1, S2, S3, S5, S8, S9, and S11; *Bacteroidota* in S4; *Bacteroidota* and *Proteobacteria* in S6 and S7; and *Firmicutes* in S10. With regard to the eukaryotic microbiota, the predominant phyla were *Basidiomycota*, *Ascomycota*, and *Cryptophyceae* in S1 and S2; *Ascomycota* and *Cryptophyceae* in S3; *Ascomycota*, *Holozoa*, and *Cryptophyceae* in S8 and S11; *Cryptophyceae* in S4 and S5; and *Holozoa* and *Cryptophyceae* in S6, S7, S9, and S10.

Principal coordinates analysis showed that the distribution of prokaryotic microbial communities in the groups was scattered, indicating that the microprokaryotic community structures in the groups were significantly different (ANOSIM: *R* = 0.52, *p* = 0.001; Figure 2C). However, no significant scattering was observed in the eukaryotic microbiota distribution (ANOSIM: *R* = 0.002, *p* = 0.925; Figure 2D).

**Figure 3:**
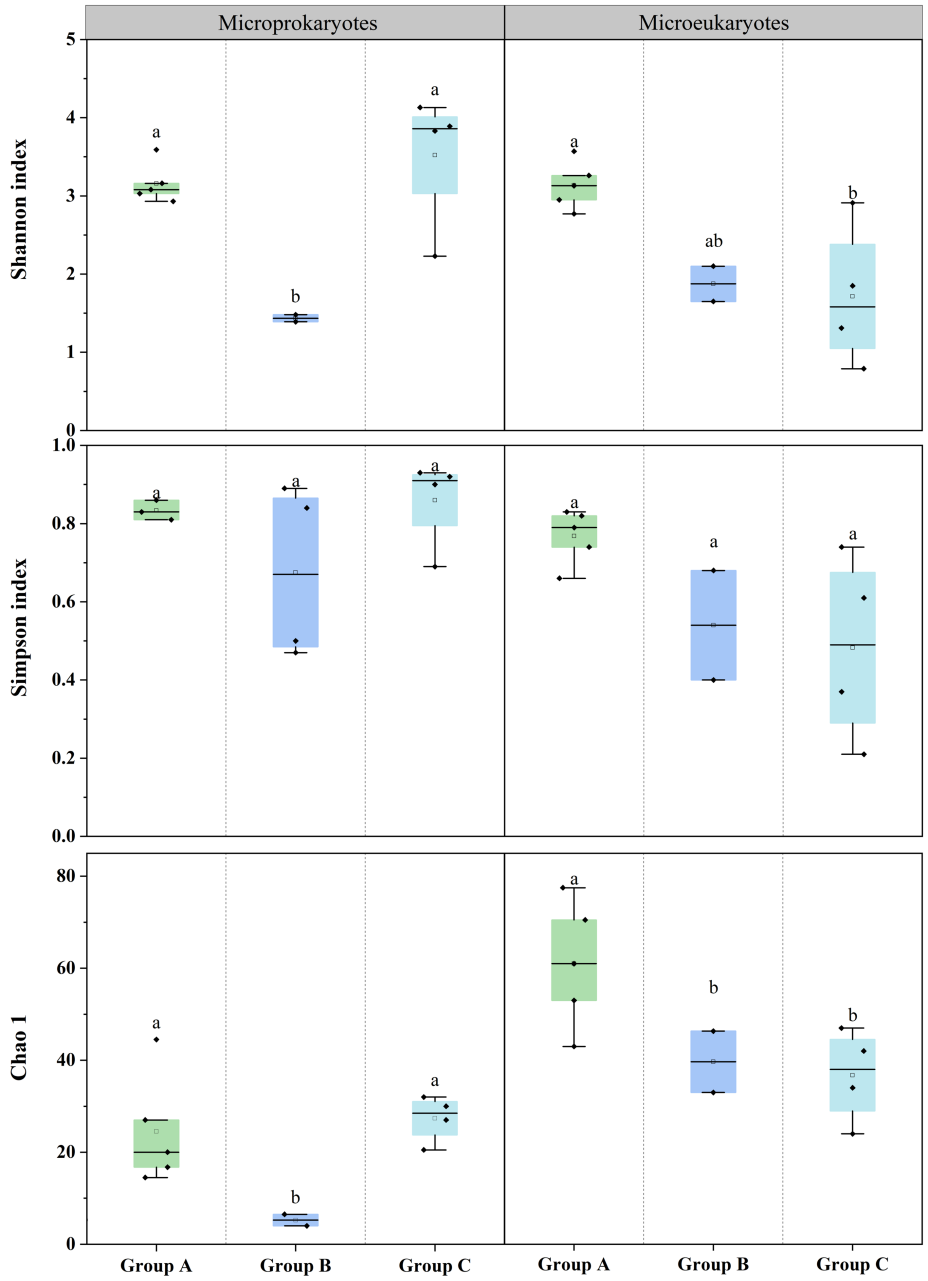
Microbial diversities at the 11 stations. Horizontal lines indicate the median, and lowercase letters above the bars indicate significant differences (*p* < 0.05) among the three groups (Fisher’s least significant difference test).

Additionally, the diversities of the prokaryotic and eukaryotic microbiota were estimated using the Shannon, Simpson, and Chao1 (Supplementary Tables S1, S2). For prokaryotic microbiota, the Shannon index ranged from 1.39 (S4) to 4.13 (S6), the Simpson’s index ranged from 0.50 (S4) to 0.93 (S6), and the Chao1 index ranged from 4 (S4) to 44.5 (S11) at the 11 stations. For the eukaryotic microbiota, the Shannon index ranged from 0.79 (S5) to 3.57 (S2), the Simpson’s index ranged from 0.21 (S5) to 0.83 (S11), and the Chao1 index ranged from 25 (S5) to 77.5 (S2) at the 11 stations. The diversity differed according to the microbial community structure. There were significant differences in the prokaryotic and eukaryotic microbiota among the three groups (Figure 3). This finding indicates that significant differences in the community structure and diversity of microprokaryotes, as well as significant differences in the diversity of microeukaryotes in Maxwell Bay.

### 3.2 Microbial distribution patterns

**Figure 4:**
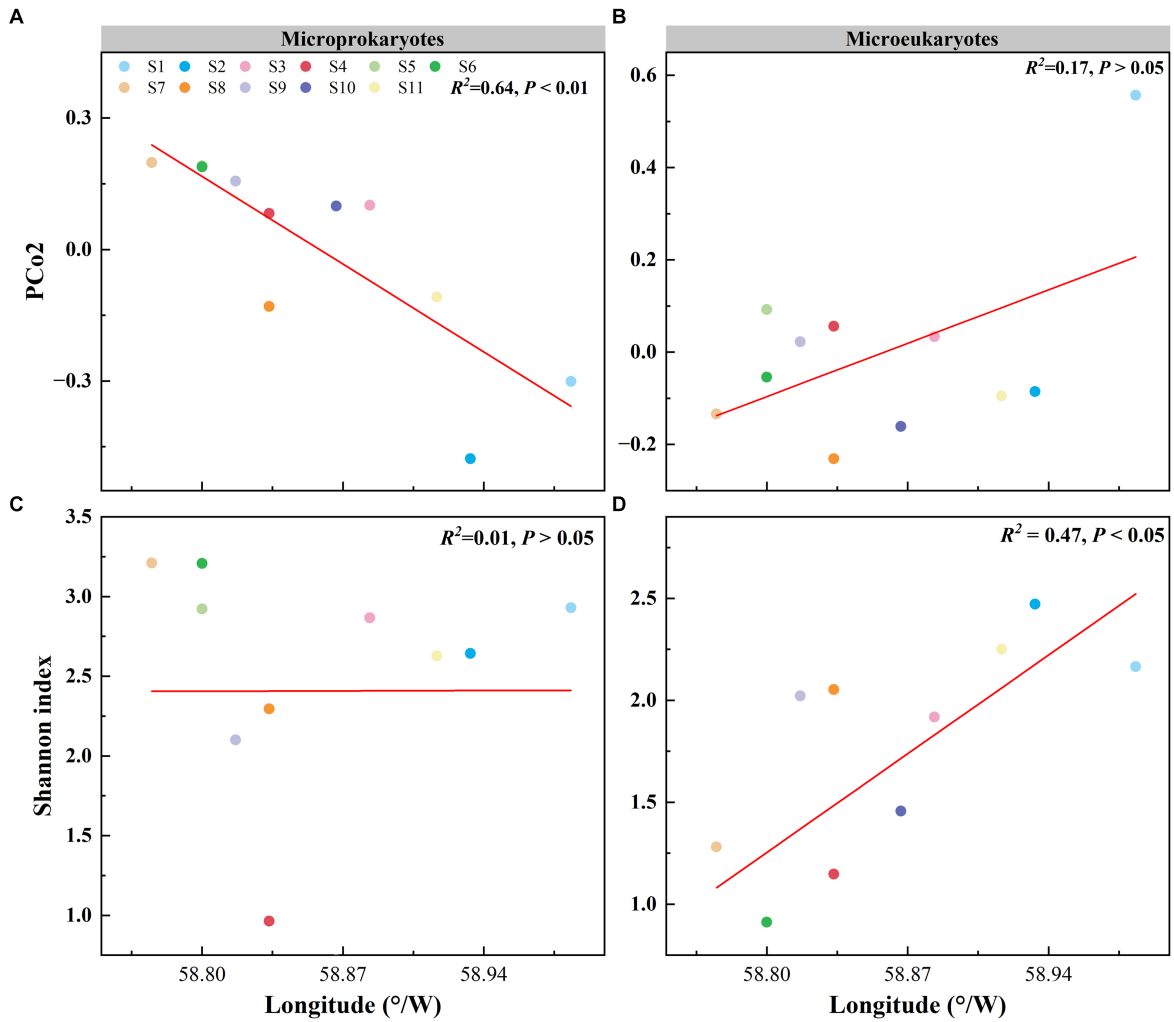
Effect of longitude on the microbial community and diversity. **(A)** Relationship between the PCo2 (microprokaryotic community) and longitude. **(B)** Relationship between the PCo2 (microeukaryotic community) and longitude. **(C)** Relationship between the microprokaryotic diversity and longitude. **(D)** Relationship between the microeukaryotic diversity and longitude.

Principal coordinates analysis of the microbial community structure revealed that the points were distributed along PCo2 (Figures 2C,D). PCo2 was subjected to correlation analysis with latitude and longitude for microprokaryotes and microeukaryotes across the 11 stations. There was a significant correlation between PCo2 and longitude in microprokaryotes (microprokaryotes: *p* < 0.01, *R^2^* = 0.64; microeukaryotes, *p* > 0.05, *R^2^* = 0.17). However, this was not observed with latitude (*p* > 0.05; Figures 4A,B; Supplementary Figure S1). This finding revealed a significant correlation between microprokaryotic communities and longitude.

In addition, the relationship between microbial diversity and latitude and longitude was analyzed. The diversity of microeukaryotes was significantly correlated with longitude, whereas no correlation was seen for microprokaryotes (microprokaryotes *p* > 0.05; microeukaryotes, *p* < 0.01, *R^2^* = 0.47; Figures 4C,D; Supplementary Figure S2). There was no significant correlation between the diversities of microeukaryotes and microprokaryotes and latitude (Supplementary Figure S3). This finding indicates that microeukaryotic diversity is more susceptible to longitudinal influence.

**Figure 5:**
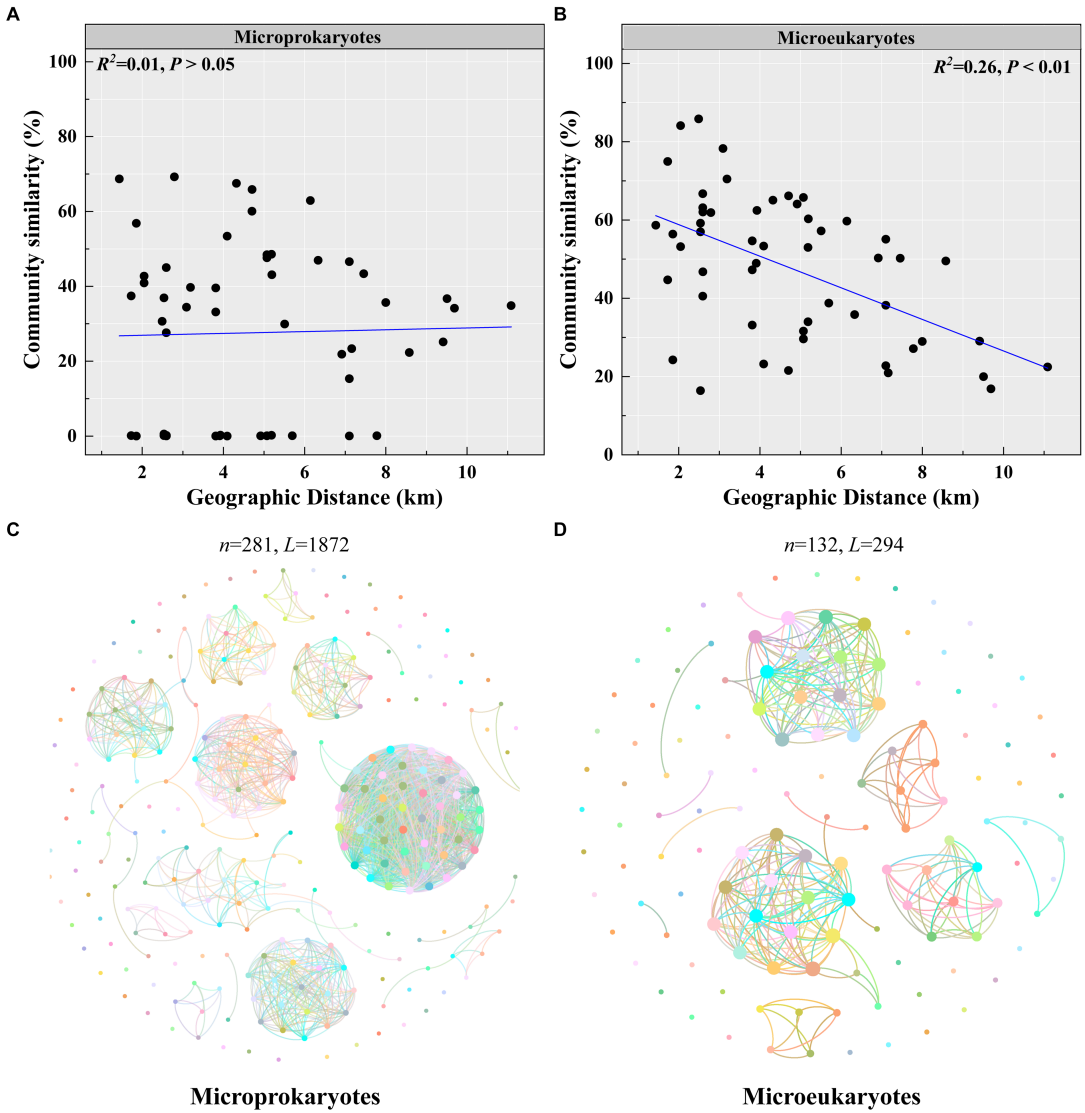
Microbial community stability. Distance-decay relationships of **(A)** microprokaryotic and **(B)** microeukaryotic (based on Bray–Curtis distance) communities. Co-occurrence networks analysis for **(C)** microprokaryotic community. **(D)** Co-occurrence networks analysis for microeukaryotic community.

Second, microeukaryotic community similarity decreased significantly with increasing geographic distance (microprokaryotes, *p* > 0.05; microeukaryotes, *p* < 0.01, *R^2^* = 0.26; Figures 5A,B). This observation reveals that the impact of spatial factors on microeukaryotes is more significant than that on microprokaryotes. Furthermore, microprokaryotic and microeukaryotic co-occurrence networks were constructed based on Spearman’s correlations among OTUs to investigate microbial stability (Figures 5C,D). A total of 281 nodes linked by 1,872 edges comprised the microprokaryotic community network, and 132 nodes linked by 1,872 edges comprised the microeukaryotic community network. These results showed that the microprokaryotic community was more complex and stable than the microeukaryotic community. All results suggest that the microeukaryotic community is more susceptible to disturbances at the 11 stations in Maxwell Bay.

### 3.3 Effects of environmental variables on shaping the microprokaryotic diversity patterns

**Figure 6:**
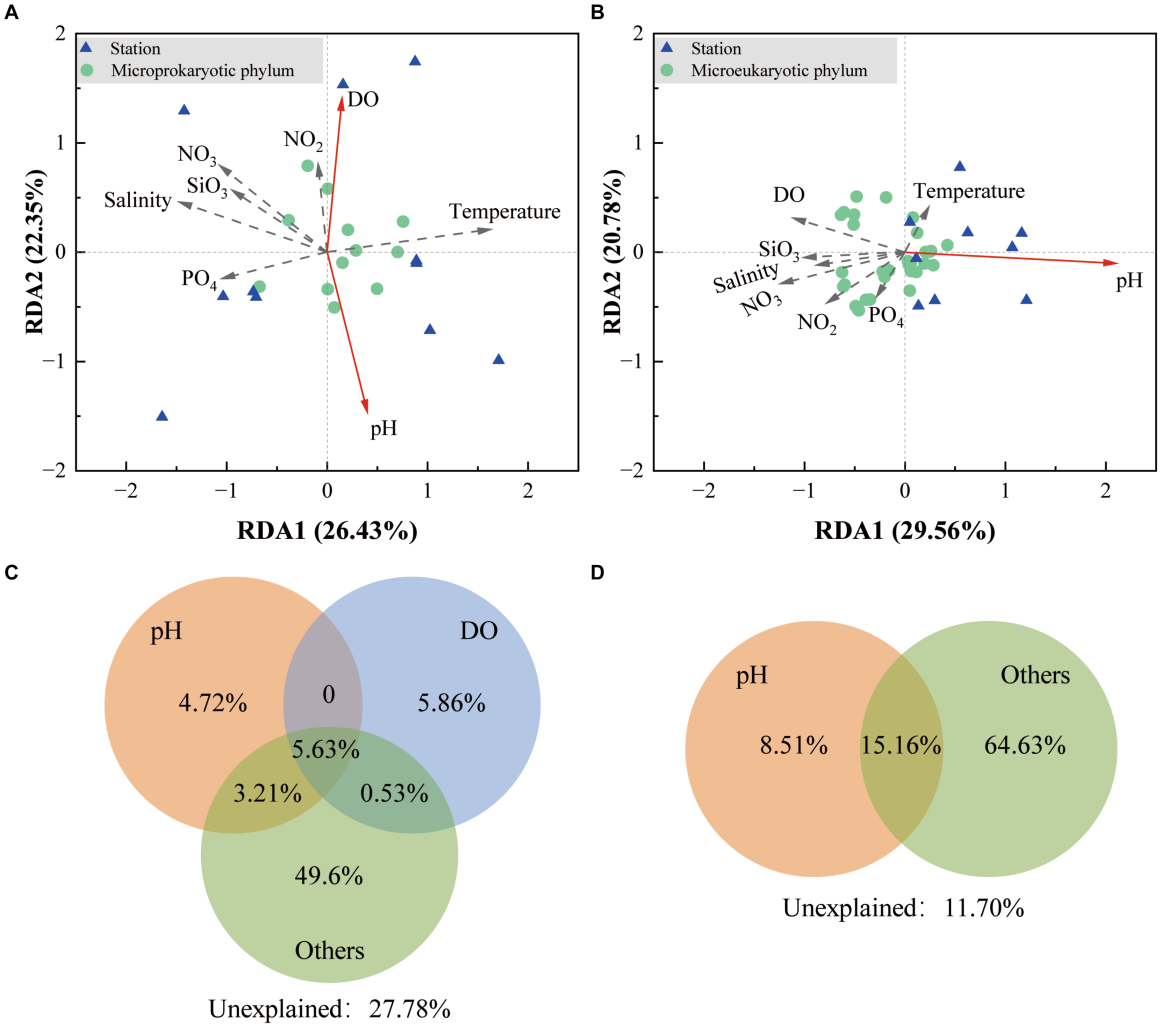
Effect of environmental factors on the microbial community. Redundancy analysis is based on environmental factors in the **(A)** microprokaryotic and **(B)** microeukaryotic communities. The green dots represent the microbial phylum; the blue triangles represent the 11 stations; the red arrows point to the different environmental factors. The solid lines indicate significant (*p* < 0.001) correlations between environmental factors and microbial communities, and the dotted lines indicate no significant (*p* > 0.05) correlations. Variance partitioning analysis showing contributions of environmental factors to the **(C)** microprokaryotic and **(D)** microeukaryotic community.

Redundancy analysis demonstrated that the first two sequencing axes revealed 26.43 and 22.35% of the microprokaryotic and 29.56 and 20.78% of the microeukaryotic community variations, respectively (Figures 6A,B). The Monte Carlo analysis established that DO and pH were environmental factors that significantly affected the microprokaryotic community (*p* < 0.05), whereas only pH significantly affected the microeukaryotic community (*p* < 0.05). Variance partitioning analysis was used to analyze crucial environmental factors. For the microprokaryotic community, pH and DO revealed 13.56 and 12.02% of community variabilities, respectively, among which 4.72 and 5.86% were independently revealed by pH and DO, respectively (Figure 6C). For the microeukaryotic community, DO revealed 23.67% of community variability, 8.51% of which was independently revealed by DO (Figure 6D).

### 3.4 Potential function of the microbial community

**Figure 7:**
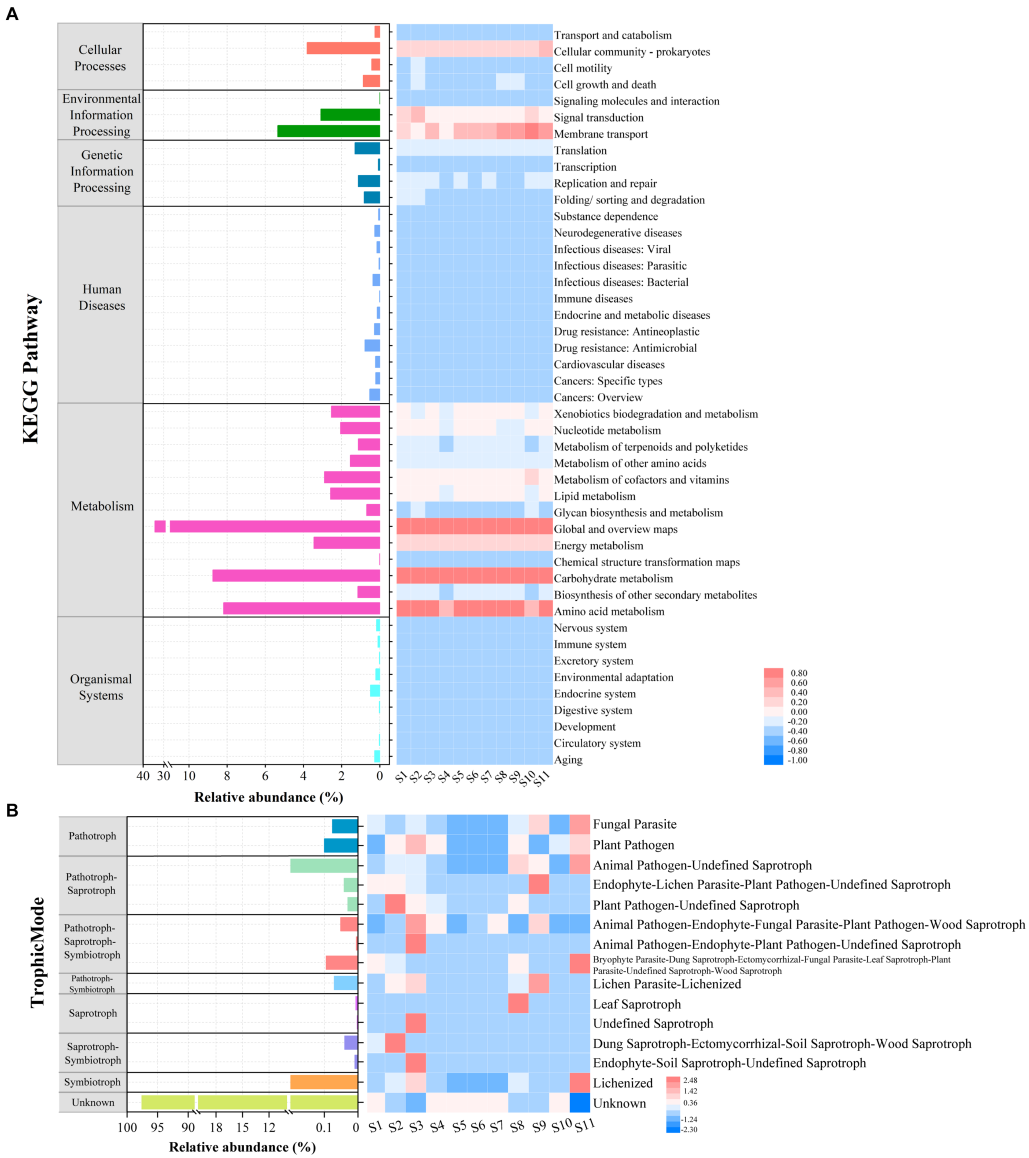
Functional prediction of microbial communities at the 11 stations. **(A)** Relative abundance of microprokaryotic potential functional categories obtained using Tax4Fun2. **(B)** Relative abundance of microeukaryotic potential functional guilds generated using FUNGuild analysis.

For the microprokaryotic community, six metabolic pathways, including 45 Kyoto Encyclopedia of Genes and Genomes (KEGG) second-classification functional pathways, were detected using Tax4Fun2. In the global and overview maps, had the highest average relative abundance (34.29%), followed by carbohydrate metabolism (8.75%), amino acid metabolism (8.20%), membrane transport (5.35%), cellular community–prokaryotes (3.81%), energy metabolism (3.46%), signal transduction (3.09%), metabolism of cofactors and vitamins (2.90%), lipid metabolism (2.59%), xenobiotics biodegradation and metabolism (2.54%), nucleotide metabolism (2.06%), metabolism of other amino acids (1.54%), translation (1.30%), biosynthesis of other secondary metabolites (1.15%), metabolism of terpenoids and polyketides (1.13%), replication and repair (1.13%), and others with average relative abundance of <1% (Figure 7A). Among them, carbohydrate and amino acid metabolisms are essential in the marine carbon and nitrogen cycles, respectively.

Based on the fungal guild classification identified using FUNGuild, eight trophic modes were detected in the microeukaryotic community (Figure 7B). The trophic modes comprised pathogens (0.18%), pathogen-saprotrophs (1.04%), pathogen-saprotroph-symbiotrophs (0.15%), pathogen-symbiotrophs (0.07%), saprotrophs (0.0090%), saprotroph-symbiotrophs (0.05%), symbiotrophs (0.92%), and unknown (97.58%). The 15 guilds were annotated. Among these, the most average abundant guild was unknown (97.58%), followed by animal pathogen-undefined saprotrophs (0.97%), lichenized (0.92%), plant pathogens (0.1%), and others with an average relative abundance of <0.1%.

## 4 Discussion

In this study, we investigated the microbial structure and diversity in Maxwell Bay using high-throughput sequencing, uncovering key patterns and environmental influences that shape these communities. Our findings revealed that the dominant microprokaryotic phyla-Proteobacteria, Bacteroidetes, and Cyanobacteria-exhibited distinct distributions corresponding to different coves (S2, S11, and S7). This is in alignment with previous studies (Kim et al., 2020; Luo et al., 2015; Zeng et al., 2014). Importantly, the sensitivity of Proteobacteria and Bacteroidetes to temperature fluctuations (Newsham et al., 2019; Prekrasna et al., 2022) suggests a strong link between these microbial communities and the unique climate of Antarctica, reinforcing their role as bioindicators of environmental change.

For the microeukaryotic community, we identified Cryptophyta, Ascomycota, and Basidiomycota as the dominant phyla, also with distributions tied to specific coves. However, our results differ from previously published findings, which could be attributed to temporal variations leading to changes in community structure, as the recent available results related to the microeukaryotic community were published in 2015 (Luo et al., 2015).

The PCoA analysis revealed significant differences among the microbial communities of the coves. This finding suggests a significant dissimilarity between the microbial communities of the three coves (Figure 2C). The microprokaryotes and microeukaryotes in the three groups exhibited distinct patterns of dissimilarity. For microprokaryotes, both community structure and diversity were significantly affected, while for microeukaryotes, only diversity was significantly impacted (Figure 3). Previous studies have shown similar results (Zheng and Gong, 2019). And our results indicate that the coves significantly impacted microprokaryotic and microeukaryotic communities.

Space has been demonstrated to be essential in shaping microbial communities in many studies (Brown et al., 2012; Martiny et al., 2006; Zhang et al., 2020). Such as water-depth has significant impact on microbial diversity and community structure (Xu et al., 2022). In addition, space could have different impact on microbial structure and diversity, such elevation has significant effect on bacterial diversity than structure (Larsen et al., 2024). In this study, we investigated the influence of spatial factors on microbial community diversity. This investigation showed that the impact of spatial factors on microeukaryotes was greater than that on microprokaryotes, with only longitude showing a significant correlation with the microprokaryotic structure (Figures 4, 5; Supplementary Figures S1–S3). These results revealed that the microeukaryotic community was unstable and susceptible to influences. For the network analysis, previous studies revealed that network stability is strongly correlated with network complexity (Cornell et al., 2023; Shen et al., 2023; Yuan et al., 2021). Our network analysis further differentiated between microprokaryotic and microeukaryotic communities, revealing greater complexity and stability within the microprokaryotic networks. This aligns with the hypothesis that complex microbial networks enhance ecosystem resilience and functional diversity (Wagg et al., 2019), a crucial factor in the sustainability of polar ecosystems. The relative simplicity and instability of microeukaryotic networks, as observed, may reflect their vulnerability to environmental disturbances, suggesting a need for ongoing monitoring.

Environmental factors significantly influence microbial communities and their functions (Centurion et al., 2021; Louca et al., 2016). In this study, environmental factors such as pH and DO were found to have significant impacts on microbial community structure. The correlation between these factors and microbial diversity emphasizes the role of human activities, particularly near research stations, in altering local environmental conditions (Liu and Jiang, 2020). The observed variations in pH and DO, which correspond with human activity, underscore the anthropogenic pressures on these sensitive ecosystems and highlight the necessity for conservation efforts.

Polar microbes are crucial components of ecosystems and significantly affect the environment (Ji et al., 2022; Papale et al., 2020). Finally, functional predictions using Tax4Fun2 and FUNGuild revealed crucial roles for microprokaryotic communities in marine carbon and nitrogen cycles, particularly through carbohydrate and amino acid metabolism. These functions are essential for nutrient cycling in extreme environments, consistent with findings from other polar studies (Yuan et al., 2023). The diverse trophic modes within microeukaryotic communities further complicate the ecological dynamics in Maxwell Bay, indicating a complex interplay of functions that warrants deeper investigation to fully understand their ecological roles and adaptive strategies in polar ecosystems.

## 5 Conclusion

In conclusion, our study significantly advances the understanding of microbial ecology in polar regions, offering new insights into the distribution and diversity of microprokaryotic and microeukaryotic communities. We found distinct differences in their community structures, with microeukaryotic communities exhibiting greater sensitivity to environmental disturbances, as revealed by spatial correlation and network analysis. Environmental factors such as dissolved oxygen (DO) and pH were identified as key drivers for microprokaryotic communities, while pH played a crucial role in shaping microeukaryotic communities.

Our functional analysis underscored the pivotal roles of carbohydrate and amino acid metabolism in microprokaryotes and highlighted the diverse trophic strategies employed by microeukaryotes. These findings suggest that the intricate balance of these microbial communities is closely tied to environmental parameters, emphasizing the need for ongoing, long-term monitoring to track the impacts of climate change on the Antarctic microbiome.

In the future, the integration of advanced technologies such as metagenomics, metatranscriptomics, and metaproteomics will be crucial. These tools will deepen our understanding of the complex lifestyles of Antarctic microorganisms and their interactions with the extreme environment. Such research is vital for predicting the future dynamics of these ecosystems in the face of global climate change.

## Data Availability

The prokaryotic microbial and eukaryotic microbial raw sequence data were uploaded in the National Genomics Data Center (NGDC) database under the accession numbers CRA013710 and CRA013713, respectively.
