# Supplementary material for: New insights into the structure and function of microbial communities in Maxwell Bay, Antarctica
Source: Front Microbiol. 2024 Sep 4;15:1463144. doi: 10.3389/fmicb.2024.1463144 (PMC11408308; doi:10.3389/fmicb.2024.1463144)
Supplement: Supplementary file 1 [file Table_1.docx]

***Supplementary Material***

**New insights into the structure and function of microbial communities in Maxwell Bay, Antarctica**

Zheng Wang^a, b^, Zhiwei Gao^a, b^, Yong Yu^a, b, c^, Huirong Li^a, b, c^, Wei Luo^a, b, c^, Zhongqiang Ji^d^, Haitao Ding^a, b, c,^ *****

^a^ Antarctic Great Wall Ecology National Observation and Research Station, Polar Research Institute of China, Ministry of Natural Resources, Shanghai 200136, China

^b^ Key Laboratory for Polar Science, Polar Research Institute of China, Ministry of Natural Resources, Shanghai 200136, China.

^c^ School of Oceanography, Shanghai Jiao Tong University, Shanghai 200030, China.

^d^ Key Laboratory of Marine Ecosystem Dynamics, Second Institute of Oceanography, Ministry of Natural Resources, Hangzhou 310012, China.

* Corresponding author

E-mail address: [dinghaitao@pric.org.cn](mailto:dinghaitao@pric.org.cn)

**This supplementary material include:**

**Supplementary tables: Table S1-S2;**

**Supplementary figures: Figures S1–S3.**

**Supplementary tables:**

**Table S1.** Microprokaryotic Shannon, Simpson’s, and Chao1 index at the 11 stations

| Sample | Shannon | Simpson | Chao1 |
| --- | --- | --- | --- |
| S1 | 3.16 | 0.86 | 20.00 |
| S2 | 3.08 | 0.83 | 16.75 |
| S3 | 2.93 | 0.81 | 27.00 |
| S4 | 1.39 | 0.50 | 4.00 |
| S5 | 3.83 | 0.90 | 30.00 |
| S6 | 4.13 | 0.93 | 32.00 |
| S7 | 3.89 | 0.92 | 27.00 |
| S8 | 3.03 | 0.84 | 14.50 |
| S9 | 2.23 | 0.69 | 20.50 |
| S10 | 1.48 | 0.47 | 6.50 |
| S11 | 3.59 | 0.89 | 44.50 |

**Table S2.** Microeukaryotic Shannon, Simpson, and Chao1 index at the 11 stations

| Sample | Shannon | Simpson | Chao 1 |
| --- | --- | --- | --- |
| S1 | 3.13 | 0.79 | 70.50 |
| S2 | 3.57 | 0.82 | 77.50 |
| S3 | 2.77 | 0.66 | 61.00 |
| S4 | 1.65 | 0.40 | 46.33 |
| S5 | 0.79 | 0.21 | 24.00 |
| S6 | 1.31 | 0.37 | 42.00 |
| S7 | 1.85 | 0.61 | 34.00 |
| S8 | 2.95 | 0.74 | 53.00 |
| S9 | 2.91 | 0.74 | 47.00 |
| S10 | 2.10 | 0.68 | 33.00 |
| S11 | 3.26 | 0.83 | 43.00 |

**Supplementary figures:**





**Figure S1.** Effect of latitude on the microbial community. (A) Relationship between the PCo2 (microprokaryotic community) and latitude. (B) Relationship between the PCo2 (microeukaryotic community) and latitude.





**Figure S2.** Effect of longitude on the microbial diversity. (A) Relationship between microprokaryotic Simpson index and longitude. (B) Relationship between microeukaryotic Simpson index and longitude. (C) Relationship between microprokaryotic Chao1 and longitude. (D) Relationship between microeukaryotic Chao1 and longitude.





**Figure S3.** Effect of latitude on the microbial diversity. (A) Relationship between microprokaryotic Shannon index and latitude. (B) Relationship between microeukaryotic Shannon index and latitude. (C) Relationship between microprokaryotic Simpson index and latitude. (D) Relationship between microeukaryotic Simpson index and latitude. (E) Relationship between microprokaryotic Chao1 and latitude. (F) Relationship between microeukaryotic Chao1 and latitude.
